# Surgical Repair of Supracristal Ventricular Septal Defect With Combined Multivalvular Disease in an Elderly Patient: A Transaortic Approach

**DOI:** 10.1093/icvts/ivag113

**Published:** 2026-04-13

**Authors:** Shinji Kanemitsu, Satoshi Maruyama, Takato Yamasaki, Koh Kajiyama

**Affiliations:** Department of Cardiovascular Surgery, Anjo Kosei Hospital, Anjo, Aichi 446-8602, Japan; Department of Cardiovascular Surgery, Anjo Kosei Hospital, Anjo, Aichi 446-8602, Japan; Department of Cardiovascular Surgery, Anjo Kosei Hospital, Anjo, Aichi 446-8602, Japan; Department of Cardiovascular Surgery, Anjo Kosei Hospital, Anjo, Aichi 446-8602, Japan

**Keywords:** supracristal ventricular septal defect, transaortic approach, aortic valve replacement, elderly

## Abstract

**Background:** Uncorrected supracristal ventricular septal defect (VSD) in an elderly patient is rare and is often complicated by aortic regurgitation, multivalvular disease, and arrhythmias.

**Case summary:** We present an elderly female with supracristal VSD, chronic atrial fibrillation, and multivalvular disease. A transaortic approach enabled direct visualization and secure patch closure through the same aortotomy used for aortic valve replacement. Concomitant mitral valve replacement, tricuspid annuloplasty, and a Maze procedure with left atrial volume reduction were performed.

**Conclusion:** This case demonstrates the transaortic approach as an effective strategy for combined VSD and valve surgery in elderly patients.

## INTRODUCTION

Supracristal ventricular septal defect (VSD) is more prevalent in East Asian and most cases that develop aortic regurgitation are treated during childhood. Unrepaired defects, particularly in the elderly are rare and often associated with pulmonary hypertension, left ventricular volume overload, and progressive valvular disease. This defect predisposes to aortic cusp prolapse and aortic regurgitation due to shunt-related Venturi effects. In elderly patients, management is further complicated by degenerative valvular disease and arrhythmias. A transaortic approach is an effective way for adult cardiac surgeons to treat supracristal VSD and multivalvular disease.

## CASE PRESENTATION

**Figure 1.**
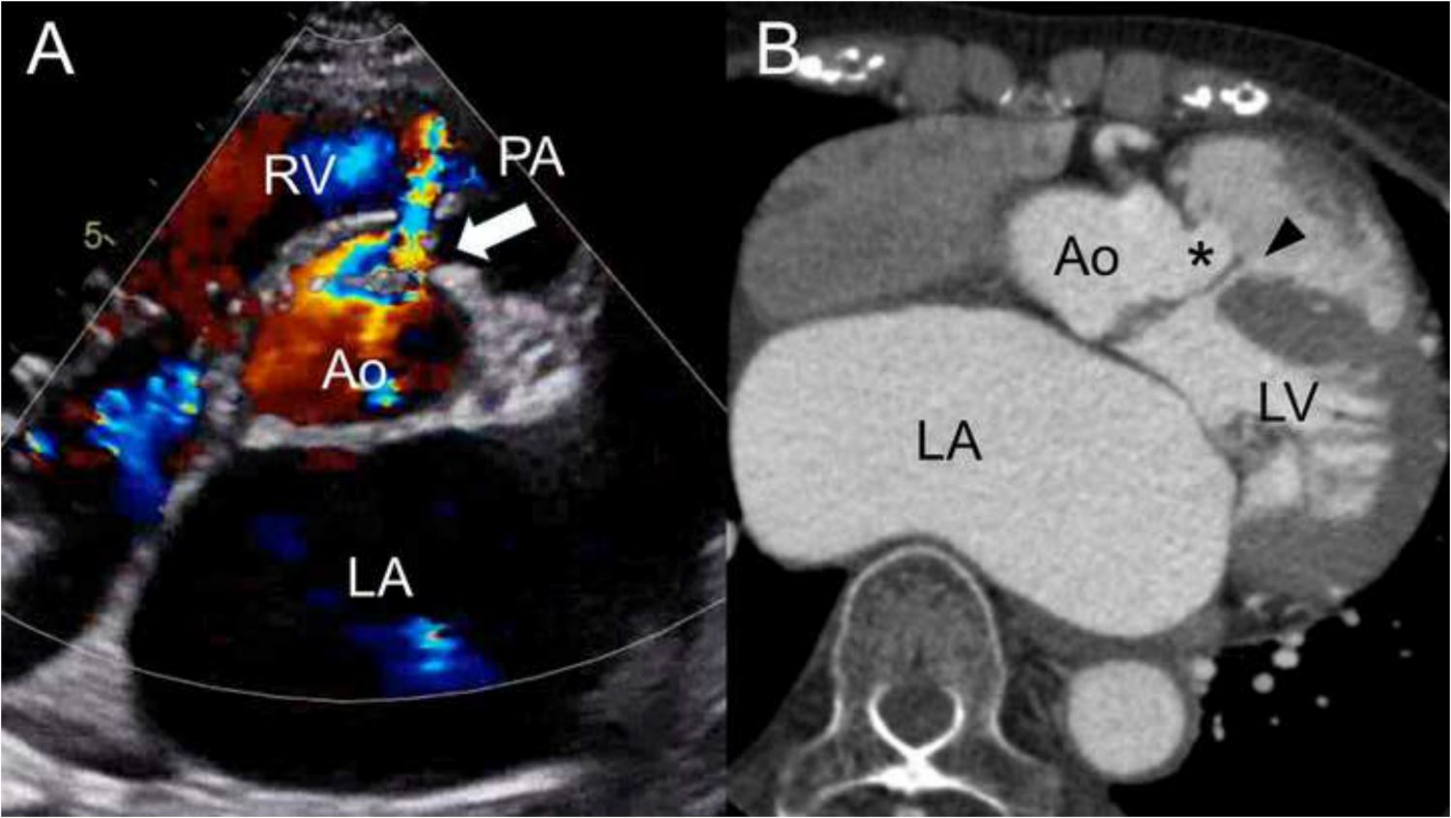
Preoperative Image of Ventricular Septal Defect. (A) Transthoracic echocardiography shows a ventricular septal defect (arrow) with a left-to-right shunt. (B) Computed tomography angiography shows right sinus of Valsalva aneurysm (asterisk) herniating into the restrictive ventricular septal defect (arrowhead) beneath the right coronary cusp, with prolapse into the left ventricle. Abbreviations: Ao = aorta; LA = left atrium; LV = left ventricle; PA = pulmonary artery; RV = right ventricle.

A 76-year-old female with a history of heart failure hospitalization was referred for surgical evaluation. She had an uncorrected VSD and chronic atrial fibrillation managed conservatively for decades. NT-proBNP levels were elevated (2446 pg/mL), but her condition improved after medical treatment. Transthoracic echocardiography revealed a 7-mm supracristal VSD beneath the right coronary cusp with significant left-to-right shunting (Qp/Qs: 2.4) (**Figure 1A**). Moderate aortic regurgitation and stenosis, mitral regurgitation, and tricuspid regurgitation were present, with marked left atrial enlargement. Computed tomography showed herniation of a right sinus of Valsalva aneurysm into the VSD (10 mm), causing malcoaptation of the aortic valve (**Figure 1B**). Given the severe volume overload and multivalvular disease, surgery was indicated.

## SURGICAL TECHNIQUE

**Figure 2.**
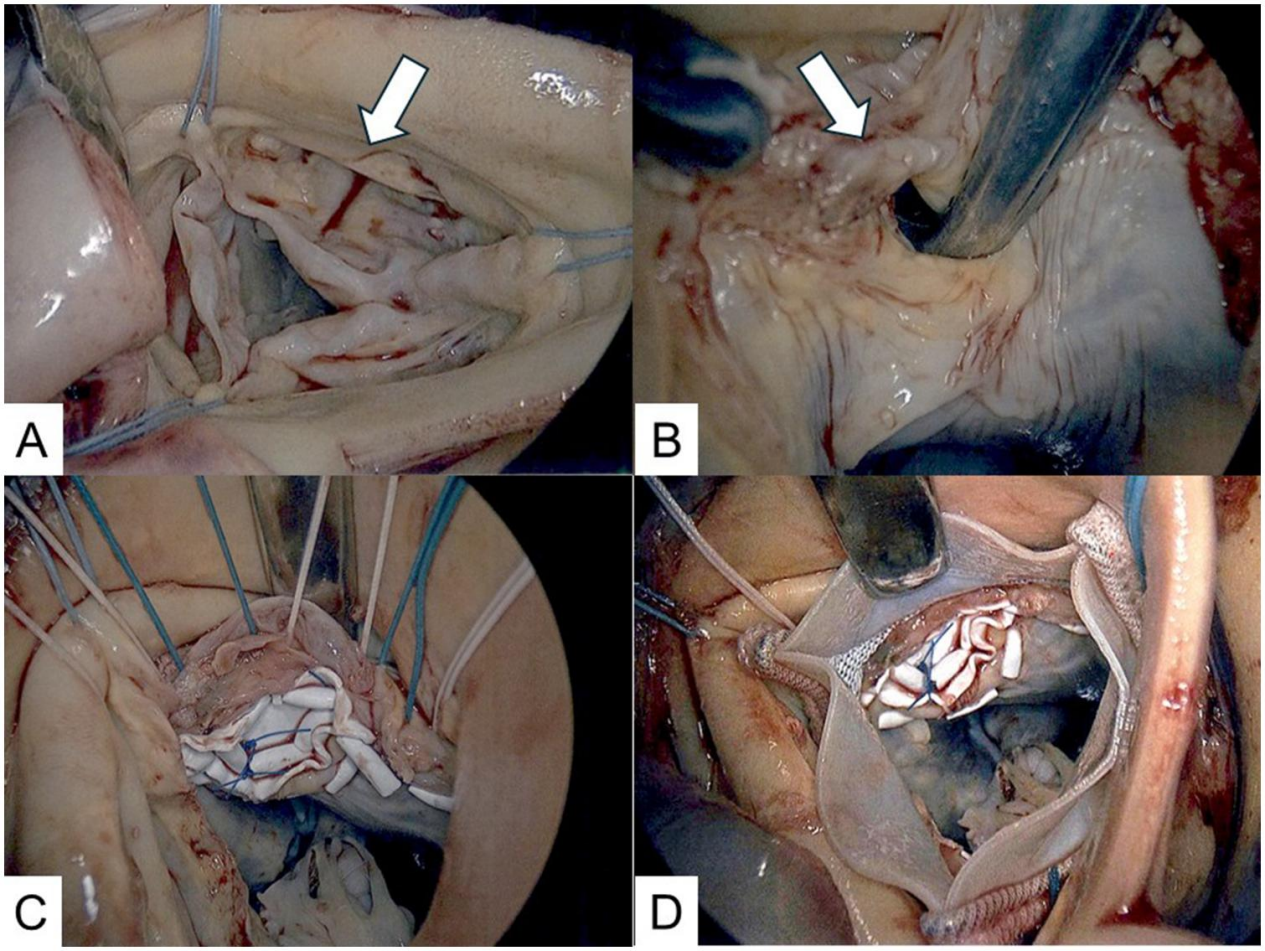
Intraoperative Findings and Repair. (A) A right sinus of Valsalva aneurysm protruding beneath the right coronary cusp (arrow). (B) Intraoperative view shows a type I ventricular septal defect (arrow) beneath the commissure the right coronary cusp. (C) The ventricular septal defect was closed and the annular portion of the patch was used as annular sutures for aortic valve replacement. (D) Aortic valve replacement with a bioprosthetic valve was performed. There was no subvalvular obstruction caused by the patch.

The operation was performed via median sternotomy. A full Maze procedure with left atrial volume reduction and excision of the left atrial appendage was performed. Due to severe sclerodegenerative changes, mitral valve replacement with a bioprosthesis (31-mm Epic, Abbott) was undertaken, followed by tricuspid ring annuloplasty. Aortotomy exposed a deformed right sinus of Valsalva aneurysm herniating into the VSD. The native aortic valve showed right coronary cusp prolapse, commissural fusion, and extensive calcification; all leaflets were excised and annular calcification debrided. An 8-10 mm supracristal VSD with muscular borders was identified beneath the commissure between the right and left coronary cusps (**Figure 2**, **Video 1**) and closed with a GORE-TEX patch. Sutures along the superior patch margin were passed through the aortic annulus and used to anchor the bioprosthetic valve (23-mm Inspiris Resilia, Edwards Lifesciences), creating an integrated “sandwich” repair. Postoperative echocardiography confirmed complete VSD closure, normal prosthetic valve function, and no paravalvular leakage. Follow-up CT showed marked reduction in left atrial volume. At 10 months post-surgery, the patient remains stable in sinus rhythm with no recurrence of heart failure symptoms.

**Video 1.**
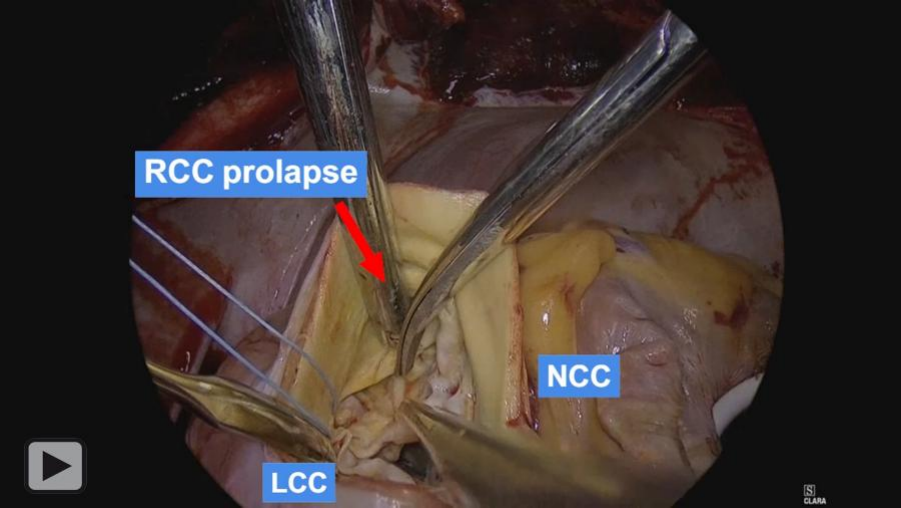
Operative Findings; the Surgical Strategy of Transaortic Simultaneous VSD Closure and Aortic Valve Replacement.

## DISCUSSION

This case highlights the feasibility of comprehensive surgical management for uncorrected supracristal VSD complicated by advanced multivalvular disease in an elderly patient. Despite advanced age, the patient’s symptoms and significant left-to-right shunt justified intervention in accordance with current adult congenital heart disease guidelines.^1^ The coexistence of a large VSD with severe mitral and aortic valve disease resulted in profound left ventricular volume overload. In supracristal VSD, aortic cusp prolapse and AR are well-recognized sequelae.^2^ In elderly patients, these congenital anomalies are often compounded by calcific aortic stenosis. Given the structural damage, AVR was selected over repair. Concomitant MVR, TAP, and surgical ablation were necessary to address all contributors to heart failure and arrhythmia in a single-stage operation. Aortic cusp prolapse with progressive AR due to the Venturi effect is a well-known consequence of supracristal VSD,^2^ and in elderly patients is frequently compounded by calcific aortic stenosis. Given the extent of structural degeneration, valve replacement rather than repair was appropriate. Concomitant mitral valve replacement, tricuspid annuloplasty, and surgical ablation addressed all major contributors to heart failure and arrhythmia.

The transaortic approach provided excellent exposure of the defect located just beneath the aortic annulus. Paediatric surgeons usually use a transpulmonary approach. However, the transaortic approach allows us to see the VSD through the aortic valve. Following aortic valve excision, direct visualization enabled precise patch placement.^3^ Integrating the VSD patch and prosthetic ring via the “sandwich” technique ensures stability and prevents paravalvular leaks. Rhythm monitoring was meticulous due to conduction system proximity. Surgical ablation for atrial fibrillation is recommended during concomitant valve surgery and can yield favourable rhythm outcomes in selected elderly patients.^4^ In cases of severe left atrial enlargement, ablation alone may be insufficient. Combining the Maze procedure with left atrial volume reduction decreases atrial wall tension and macro-reentrant circuits,^5^ likely contributing to the durable restoration of sinus rhythm in this patient.

## CONCLUSION

A comprehensive surgical strategy utilizing a transaortic approach is viable and effective for supracristal VSD and complex multivalvular disease. Transaortic simultaneous VSD closure and aortic valve replacement successfully addressed the pathophysiology with minimized invasiveness.

## Data Availability

The data underlying this article will be shared on reasonable request to the corresponding author.
